# Insights into the Pathophysiology of Psychiatric Symptoms in Central Nervous System Disorders: Implications for Early and Differential Diagnosis

**DOI:** 10.3390/ijms22094440

**Published:** 2021-04-23

**Authors:** Giulia Menculini, Elena Chipi, Federico Paolini Paoletti, Lorenzo Gaetani, Pasquale Nigro, Simone Simoni, Andrea Mancini, Nicola Tambasco, Massimiliano Di Filippo, Alfonso Tortorella, Lucilla Parnetti

**Affiliations:** 1 Section of Psychiatry, Department of Medicine and Surgery, University of Perugia, Piazzale Lucio Severi 1, 06132 Perugia, Italy; alfonso.tortorella@unipg.it; 2 Section of Neurology, Department of Medicine and Surgery, University of Perugia, Piazzale Gambuli 1, 06132 Perugia, Italy; elena.chipi@outlook.com (E.C.); federico.paolinipaoletti@gmail.com (F.P.P.); lorenzo.gaetani@unipg.it (L.G.); pasquale.nigro1987@gmail.com (P.N.); simone.simoni@unipg.it (S.S.); mancini1andrea@gmail.com (A.M.); n.tambasco@libero.it (N.T.); massimiliano.difilippo@unipg.it (M.D.F.); lucilla.parnetti@unipg.it (L.P.)

**Keywords:** central nervous system disorders, neurodegenerative diseases, neuroinflammatory diseases, psychiatric symptoms, pathophysiology, biomarkers, cerebrospinal fluid, early diagnosis, differential diagnosis

## Abstract

Different psychopathological manifestations, such as affective, psychotic, obsessive-compulsive symptoms, and impulse control disturbances, may occur in most central nervous system (CNS) disorders including neurodegenerative and neuroinflammatory diseases. Psychiatric symptoms often represent the clinical onset of such disorders, thus potentially leading to misdiagnosis, delay in treatment, and a worse outcome. In this review, psychiatric symptoms observed along the course of several neurological diseases, namely Alzheimer’s disease, fronto-temporal dementia, Parkinson’s disease, Huntington’s disease, and multiple sclerosis, are discussed, as well as the involved brain circuits and molecular/synaptic alterations. Special attention has been paid to the emerging role of fluid biomarkers in early detection of these neurodegenerative diseases. The frequent occurrence of psychiatric symptoms in neurological diseases, even as the first clinical manifestations, should prompt neurologists and psychiatrists to share a common clinico-biological background and a coordinated diagnostic approach.

## 1. Introduction

Psychiatric and neurological diseases often overlap with each other and co-occur [1]. Psychiatric symptoms may present along the course of several central nervous system (CNS) disorders, sometimes preceding the onset of typical neurological symptoms and signs [2,3]. In the clinical context, the presence of psychopathological manifestations in neurological diseases raises several issues. First, the onset of neurological diseases with psychiatric symptoms may cause misdiagnosis, thus hindering the early identification of the underlying CNS disorder and leading to inadequate treatment and worse prognosis [4,5,6]. Furthermore, the development of different psychopathological manifestations during the following stages of neurological diseases increases the clinical complexity of these conditions and affects individual functioning and the overall disease burden [7,8]. 

The occurrence of psychiatric symptoms in neurological diseases may underpin different causes [9]. In some cases, such symptoms can be reactive to the diagnosis of the CNS disorder itself [10,11,12], while in other cases they are reported as possible consequences of pharmacological treatments [13,14]. In addition, the same pathophysiological pathways underlying neurological manifestations can contribute to the development of psychopathological features in CNS disorders, strongly suggesting a neurobiological link between neurological and psychiatric symptoms [9,15,16]. 

During the last three decades, the use of diagnostic tools able to provide objective and reproducible findings supported the transition from a clinical characterization of CNS diseases to a combined framework that considers their functional, structural and biochemical correlates [17,18]. Particularly, body fluids biomarkers allow for the molecular characterization of CNS disorders according to their underlying neurobiological substrate, thus improving diagnostic accuracy and better addressing treatment approaches [19,20]. 

In this review, we will dedicate specific attention to the occurrence of psychopathological manifestations in neurological diseases including Alzheimer’s disease (AD), the behavioral variant of fronto-temporal dementia (bvFTD), Parkinson’s disease (PD), Huntington’s disease (HD), and multiple sclerosis (MS). First, we will provide an overview of psychiatric symptoms in these conditions, also focusing on brain circuits linked to their occurrence, and neuroimaging correlates of brain networks alterations. The pathophysiological mechanisms related to psychiatric symptoms in CNS diseases at neuronal and synaptic levels will be also presented. Secondly, we will focus on the role that cerebrospinal fluid (CSF) and blood biomarkers may play in the early identification of neurological diseases presenting with psychiatric manifestations, also analyzing their potential contribution to add further insight into the pathophysiology of psychiatric symptoms in CNS disorders.

## 2. Psychiatric Symptoms in the Clinical Course of Neurodegenerative and Neuroinflammatory Diseases

**Figure 1:**
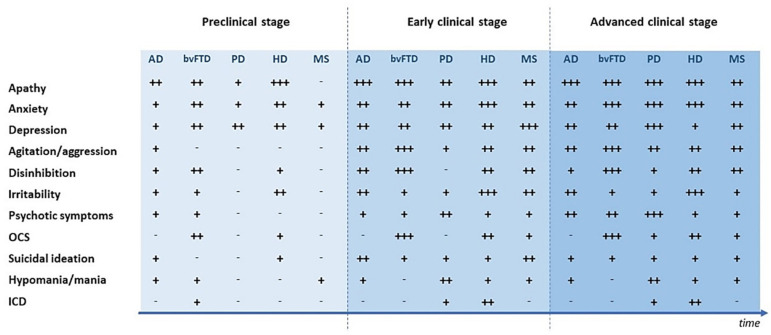
Psychiatric symptoms during different stages of neurodegenerative and neuroinflammatory diseases. AD = Alzheimer’s disease; bvFTD = Behavioral variant of fronto-temporal dementia; HD = Huntington’s disease; MS = Multiple sclerosis; PD = Parkinson’s disease; ICD = Impulse control disturbances; OCS = Obsessive-compulsive symptoms. - = Not reported; + = Prevalence <25%; ++ = Prevalence 25–50%; +++ = Prevalence >50%.

Psychiatric symptoms can appear during neurodegenerative and neuroinflammatory diseases with a variable prevalence, depending on the specific disease and the considered psychopathological manifestation [8,21,22]. Indeed, a wide range of psychopathological expressions including mood, anxiety, psychotic and obsessive-compulsive symptoms (OCS) can be observed [23,24]. Furthermore, subjects affected by CNS diseases could present with more psychiatric symptoms together, thus resulting in a more complex clinical picture [5,25]. Psychopathological features can display different levels of clinical severity, lying on a continuum that goes from subsyndromal manifestations to full-blown disorders that comply with traditional nosographic criteria [3,26]. Psychiatric symptoms occur at any stage of CNS disorders, and clinical pictures may vary along the disease course [27,28,29]. Additionally, despite the fact that different neurological conditions share common psychiatric features, psychopathological manifestations can show distinct symptom clusters depending both on disease and individual expression [21,30], which makes the overall clinical picture even more challenging to interpret. Specific psychopathological features occurring in neurodegenerative and neuroinflammatory diseases, with particular attention to their prevalence and to their onset stage, are described in Figure 1.

### 2.1. Psychiatric Symptoms in the Preclinical Phase of Neurological Diseases 

In this review, we will refer to the period before the typical clinical picture of neurological diseases becomes manifest as to “preclinical phase”. During this phase, psychiatric symptoms may frequently occur. Depressive symptoms are reported in AD prior to mild cognitive impairment (MCI) with a prevalence of about 20% [28,31], as well as in preclinical bvFTD, where a prevalence up to 40% has been described [23,32]. Depression during the preclinical stages of AD and bvFTD can be often associated with other affective symptoms and mood changes [15,33]. Similarly, affective symptoms are already present at the time of diagnosis in PD and HD in a percentage of cases up to 30%, and may precede neurological symptoms of several years [5,34,35]. Noteworthy, depressive and anxiety symptoms are listed among clinical markers of prodromal PD in the most updated diagnostic criteria [36,37]. The lack of an accurate evaluation of psychiatric symptoms, i.e., insufficient attention dedicated to their late-life onset and to a negative personal psychiatric history, often leads to the diagnosis of a primary psychiatric disorder in these subjects [15,38]. As a consequence, inappropriate psychopharmacological treatment is frequently prescribed, further affecting the clinical presentation, and contributing at the same time to the lack of adequate medical care for the underlying neurological condition [33,39]. As for neuroinflammatory diseases, psychiatric symptoms in MS are reported to be more prevalent after the occurrence of neurological symptoms, but specific symptomatologic clusters have also been identified prior to the diagnosis of MS [40,41]. A pure psychiatric presentation of MS is reported in about 2–3% cases [40,42], but depression and anxiety may precede the onset of neurological symptoms by about 10 years in up to 20–25% subjects [43,44,45], and have been hypothesized to be part of a MS prodrome [41]. Manic or hypomanic features, including elevated mood, increased energy, disinhibition and impulsivity, may also precede neurological manifestations, usually underpinning a comorbidity between MS and bipolar disorders [22,46]. 

### 2.2. Psychiatric Symptoms in the Early Clinical Stages of Neurological Diseases

During the early stages of neurological diseases, psychiatric manifestations can show different courses. Indeed, they may present in some cases a progressively increasing trend during the first years after the diagnosis, whilst in others they can decrease or remain stable over time [6,47]. Among psychiatric symptoms, apathy frequently occurs during early clinical stages, and it remains significantly prevalent also during advanced stages [48]. Apathy is characterized by multidimensional symptoms and can define a clinical syndrome per se, even in the absence of co-morbid depression [25,29,49]. In early AD, apathy shows a prevalence that reaches 70% [50,51] and is more typically characterized by the loss of motivation and executive dysfunction [52,53], whilst in bvFTD apathy occurs in up to 80% cases [32,54], presenting itself with a more frequent emotional profile [55]. Apathy also occurs in more than one third of subjects affected by PD and in a half of those with HD, typically leading to difficulties in organization and planning in these populations [56,57,58]. Apathy may have a prognostic value in all of the considered neurodegenerative diseases, since it seems to predict a faster cognitive decline [5,25,55,59]. In MS, depressive symptoms occur with a prevalence of 50% or higher during the years that immediately follow the diagnosis [7,60,61]. The onset of depression may be vague and involve clinical features that are frequently misinterpreted as typical MS symptoms, namely fatigue and sleep, appetite loss, memory and concentration disturbances [22]. The possible under-diagnosis of depressive symptoms in MS may contribute to the reduction of health-related quality of life, and to the occurrence of further complications, such as suicidal ideation [30].

### 2.3. Psychiatric Symptoms in the Advanced Clinical Stages of Neurological Diseases 

At later stages of CNS disorders, psychiatric symptoms may be more difficult to recognize, due to their overlap with progressive functional and cognitive decline [6]. Agitation and aggressive behaviors represent frequent symptoms in the advanced stage of AD and bvFTD, and significantly contribute to serious impairment in social interactions [8,62]. Moreover, psychosis frequently appears in middle and advanced stages of AD, mainly consisting of non-bizarre paranoid delusions and hallucinations, reaching a prevalence of 40% [63]. In bvFTD, OCS are also frequent, occurring in up to 60% of patients at later stages [32,64], and are more often represented by complex compulsive behaviors, while obsessions are less prevalent [65]. 

Another relevant issue concerning psychiatric symptoms in CNS disorders is their potential association with medications. For instance, in PD dopaminergic treatment is reported as a possible cause of psychosis, represented by non-threating, well-formed, simple hallucinations, which more frequently occur after treatment with high dosage dopamine agonists [27,66,67]. Further symptoms that may be associated with drugs acting on the dopaminergic system are impulse control disturbances (ICD), such as gambling and compulsive shopping, and impulsive behaviors, i.e., hypersexuality and hoarding, with the reported prevalence reaching >20% [29,68]. 

Psychiatric symptoms during later stages of neurological diseases could entail several difficulties in assessment [6]. This could be the case of depression during advanced HD, that is not reported as frequent but is hypothesized to be under-estimated [5]. On the other side, irritability reaches a prevalence of >70% and is characterized by the frequent co-occurrence of problems in controlling anger, as well as by agitation/aggression and disinhibition [69]. As for MS, anxiety represents a prevalent clinical feature in the advanced stages, with a particularly higher frequency of anxiety-related somatic symptoms in patients with longer disease duration [2,70]. Similarly to what has been reported for depression, somatic symptoms of anxiety, i.e., fatigue and muscle tension, represent clinical features that may be misunderstood as typically connected to the neuroinflammatory disease, in the absence of specific tools that could allow for a clearer characterization of these symptoms in MS [71].

## 3. Brain Circuits Involved in Psychiatric Symptoms Occurring in CNS Disorders 

Multiple brain circuits dysfunctions are thought to contribute to the pathophysiology of psychiatric symptoms in CNS disorders, and to regulate their manifestations. These networks can be investigated by means of techniques measuring neuronal oscillations (i.e., electroencephalogram, EEG; magnetoencephalography, MEG) and brain imaging [72,73,74,75,76,77,78,79,80,81,82,83]. Specifically, the methodological advances in brain imaging (i.e., new techniques, increased spatiotemporal resolution, improved image post-processing, and analysis strategies) opened the way for a deluge of studies reporting functional and structural changes underlying psychiatric symptoms in neurodegenerative and neuroinflammatory diseases. For this reason, in this review we will focus on neuroimaging correlates of brain networks alterations.

### 3.1. Alzheimer’s Disease

Increasing evidence indicates that psychiatric symptoms in AD are associated with neurodegeneration affecting specific brain circuits [82,84,85]. Networks recruiting the frontal cortex, anterior cingulate cortex (ACC), and insula have been considered involved in the pathogenesis of apathetic behavior [82,86]. Specifically, structural imaging studies reported decreased integrity and volume loss both in ACC and frontal cortex [87,88]. Moreover, in resting state functional magnetic resonance imaging (rs-f-MRI) analysis, AD patients with apathy reported default mode network (DMN) dysfunction [83,84,89]. DMN is a set of brain regions with high degrees of functional connectivity (FC), including medial prefrontal cortex, posterior cingulate cortex (PCC), precuneus, inferior parietal lobule, lateral temporal cortex, and hippocampal formation, which is crucial for the consolidation of autobiographical memory and for the processing of emotionally-salient stimuli [90,91]. 

A decreased FC in the DMN has also been proposed as an important factor in the emergence of depression in AD patients [92,93]. Structural MRI studies found that depressive symptoms resulted associated with AD-related brain changes, such as reduced entorhinal cortical thickness and with decreased gray matter volumes in left middle frontal cortex [94,95,96]. Interestingly, in patients with MCI due to AD, depressive symptoms were associated with progressive atrophy of frontal regions, also being reported as an additional risk factor for conversion to dementia [97]. 

Dysfunctions in brain circuits associated with emotional regulation, such as the salience network (SN), may reduce the capacity to process and regulate behaviors properly [98] and have thus been considered to be potentially involved in the onset of agitation in AD. The SN is a large-scale network involving as principal hubs the anterior insula (AI) and the dorsal ACC, connected to several limbic areas and subcortical structures, playing a role in the self-regulation of behavior by the integration of sensory, emotional, and cognitive information. Structural alterations in several brain regions that are implicated in the AD pathogenesis, such as frontal cortex, PCC, and hippocampus, have also been consistently associated with agitation [99].

### 3.2. Behavioral Variant of Fronto-Temporal Dementia 

Patients with bvFTD showed reduced FC of the SN developed from an initial fronto-insular degeneration [100,101]. Moreover, connectivity reductions observed in frontal and temporal regions of DMN may underlie deficits in meta-cognitive processes and insight [102,103]. Most structural studies in bvFTD focused on apathy, as one of the most frequent psychiatric symptoms. For example, a study found that grey matter loss severity in frontal, temporal, and limbic structures was correlated with apathetic behavior [104]. Furthermore, apathy severity showed association with cortical thinning of the lateral parts of the right-sided frontal, temporal and parietal lobes [105]. Concerning diffusion tensor imaging (DTI), apathy correlated with reduced fiber integrity in inferior fronto-occipital fasciculus and forceps minor, reported as decreased fractional anisotropy (FA) values [106]. 

### 3.3. Parkinson’s Disease 

The variety of different psychiatric symptoms occurring in PD reflects the involvement of distinct brain circuits. A dysregulation of frontostriatal and mesocorticolimbic dopaminergic circuits, already associated with the onset of depressive disorders and bipolar disorders [107], has been suggested to play a key role in the onset of depression during prodromal and early PD [108,109]. Alterations of FC, studied by rs-f-MRI, were specifically observed in the pathway from bilateral anterior insula and posterior orbitofrontal cortices to right basal ganglia [74]. Moreover, diffusion MRI connectometry, an advanced analytic approach for local connectivity, showed significant differences in the left and right uncinate fasciculi, left and right inferior longitudinal fasciculi, left and right fornices, left inferior fronto-occipital fasciculus, right corticospinal tract, genu of corpus callosum, and middle cerebellar peduncle when comparing depressed and non-depressed PD patients [110]. 

In PD patients with anxiety, decreased intrinsic connectivity within the DMN and sensorimotor network (SMN), increased connectivity within the executive-control network (ECN), and divergent connectivity measures within SN and frontoparietal networks (FPN) were detected [111]. In a multimodal imaging study, PD patients with anxiety showed clusters of cortical thinning in the bilateral frontocingulate and left parietal cortices and higher internetwork resting-state FC between the “fear circuit” and SN was reported [112]. 

Pathological mechanisms underlying apathy in PD may involve complex circuitry dysfunctions [58,113,114]. In a multimodal imaging study, subclinical symptoms of apathy were associated with increased white matter axial and mean diffusivity and with decreased frontostriatal and frontolimbic FC [75]. The amplitude of low-frequency fluctuations (ALFF), indicator of regional intensity of spontaneous fluctuations in the blood oxygenation level dependent (BOLD) signal, decreased significantly in the bilateral nucleus accumbens, dorsal ACC, and left dorsolateral prefrontal cortex in PD patients with apathy compared to healthy controls [115]. 

Similar circuits dysfunctions found in apathetic behavior seem to be associated with ICD [116,117]. Specifically, PD patients with ICD showed reduced activation in regions that play an important role in risk evaluation, impulse control and response inhibition such as the ventral striatum, ACC and orbitofrontal cortex, as well as dysfunctions of the reward circuits [108,118]. 

### 3.4. Huntington’s Disease

The relationship between the disruption of brain networks and psychiatric symptoms in HD is still poorly understood. Psychiatric symptoms have been primarily attributed to altered connecting circuitry between subcortical areas like basal ganglia and frontal lobes [5,119]. Specifically, the degeneration of the striatum and the orbitofrontal-subcortical circuit appeared to be associated with the development of socially inappropriate behaviors [120], while the complex cortico-subcortical network was linked to the emergence of apathy [121]. In morphological MRI studies, a smaller volume of the thalamus showed a higher probability of the presence of apathy in pre-HD [122], while reduced caudate and putamen volumes were significantly related to apathy scores in prodromal HD [123]. DTI revealed that interindividual variability in the disruption of corticostriatal tracts might explain the heterogeneous severity of apathy profiles [124]. 

Correlations between depressive symptoms with increased FC (rs-f-MRI) and decreased structural connectivity (tractography) were found for connections in the DMN and basal ganglia of pre-HD patients [125]. In a combined DTI and voxel-based morphometry (VBM) study, DTI revealed lower FA values in the frontal cortex, ACC, insula and cerebellum of the pre-HD group with subthreshold depressive symptoms, when compared with the non-depressed pre-HD group; in contrast, VBM measures were similar in both groups [126]. 

### 3.5. Multiple Sclerosis 

In MS, the immune system dysregulation and the involvement of myelinated axons leading to white matter disconnection mechanisms negatively affect brain networks, with a consequent impact on disease progression, cognitive functioning, and behavioral aspects [127]. Pathological changes in both CNS white matter and grey matter structures could play a pivotal role in the pathogenesis of MS-related psychiatric symptoms [128,129,130]. Most functional and structural studies on MS focused on depression. Disruptions in white matter tracts connecting limbic structures result in altered connectivity between core components of emotional networks and may conduct to abnormal processing of emotional stimuli [131], which might explain the high prevalence of depression in MS patients [132,133,134,135,136]. Higher lesion load in the left arcuate fasciculus, prefrontal cortex, anterior temporal lobe, and parietal lobe were also found to be associated with depression in MS patients [137]. Moreover, depression was associated with cortical atrophy of regions located in the bilateral frontal lobes, in particular of the prefrontal cortex, contributing to dysfunctional coping strategies that might promote the development of depressive symptoms [138,139]. Furthermore, a significantly lower Vermis Crus I volume and a reduced olfactory bulb volume were detected in depressed MS patients when compared to non-depressed ones [61,62]. 

### 3.6. Future Perspectives

Different imaging techniques may help elucidating processes underlying psychiatric symptoms in neurological diseases at different levels of pathology. A promising avenue for future research could be the widespread use of advanced MRI techniques, such as fMRI, and the implementation of multimodal imaging, as a potential biomarker of psychiatric symptoms in prodromal CNS disorders.

## 4. Molecular and Electrophysiological Correlates of Synaptic Dysfunction Underlying Psychiatric Symptoms in CNS Disorders

Beyond macroscopic and MRI-detectable structural damage, brain network dysfunction may also rely on the presence of microstructural and functional abnormalities at the neuronal and synaptic level. Neural plasticity in the human cortex and subcortical structures involves the reorganization of synaptic connections in an effort to model brain circuits to preserve (or delete) traces of learning processes, changing environments, and pathological insults. Synapses can be either potentiated (long-term potentiation, LTP) or weakened (long-term depression, LTD, and depotentiation) to guarantee homeostatic processes and define the connection maps underlying normal brain functioning [140,141,142]. Such physiological synaptic mechanisms, and specifically brain plasticity, have been clearly shown to be altered during several human neurological diseases [129,143,144,145,146]. 

### 4.1. Synaptic Dysfunctions in CNS Disorders

A failure of plastic modelling of neural networks has been demonstrated both in the hippocampus/medial temporal lobe and in deep basal ganglia structures in experimental models of dementia [147,148,149], movement disorders [150,151,152,153,154,155,156], and neuroinflammatory diseases [157,158,159,160,161]. Interestingly, alterations were shown not only in the synaptic ability to strengthen relevant connections, but also in the possibility to reverse previously induced synaptic potentiation [155,162,163,164,165], thus impairing the removal of unnecessary memories and leading to abnormal information storage in brain networks. 

Clinical studies confirmed the presence of altered brain plastic properties and intracortical transmission in patients with AD [166,167,168,169], FTD [170,171], PD [172,173,174], HD [175,176,177], and MS [178,179,180], hypothesizing that the inability to set proper synaptic weights in brain networks may drive the onset of disease symptoms even before the occurrence of irreversible structural damage. According to these studies, in all these conditions the final event would be represented by loss of plasticity and altered network dynamics, although the pathogenic trigger may significantly differ. Several neuropathological changes have been indeed demonstrated to interfere with synaptic function, including, but not limited to, tauopathy, amyloidosis, and synucleinopathy [149,150,151,152,181,182,183]. Moreover, in the same brain structures, synaptic transmission and plasticity can be disrupted through immune-dependent pathways associated with neuroinflammatory changes, such as activation of innate or adaptive immunity and release of soluble proinflammatory mediators [129,184].

### 4.2. Synaptic Dysfunctions and Psychiatric Disorders

Synaptopathy and the subsequent dysfunction of neural network dynamics are thought to underlie not only the pathogenesis of neurological diseases, but also of psychiatric disorders. In the early 1900s Sigmund Freud already hypothesized that an alteration of the experience-dependent mechanisms shaping brain networks may be involved in the pathogenesis of mental disorders, describing the neural processes underlying conscious and unconscious memory long before the description of synaptic plasticity [185]. To date, many clinical reports support this view, since synaptic plastic properties have been found altered in several psychiatric conditions such as Major Depressive Disorder (MDD) [186,187], bipolar disorders [188,189] and schizophrenia [190,191]. Preclinical studies further support the relevance of dysfunctional synaptic transmission in mental disorders, suggesting possible novel synapto-centric therapeutic approaches [192,193,194,195]. 

Thus, synaptic dysfunction can be seen as a common downstream event at the border zone between neurology and psychiatry. In this scenario, experimental models represent important tools for the identification of the pathways involved in the synaptic pathogenesis of psychiatric symptoms in neurological diseases. For instance, evidence derived from preclinical studies suggests that the non-physiological rescue of striatal synaptic plasticity induced by dopamine-replacement therapies may participate in the pathogenesis of ICD during PD [196]. The loss of the GABAergic tonic inhibition of striatal medium spiny neurons has been proposed as trigger event for obsessive-compulsive-like behavior in an experimental model of HD, suggesting that a dysfunction of cortico-striato-thalamo-cortical transmission is involved in the pathogenesis of psychiatric symptoms in this disease [197]. In this context, it should also be noted that an altered striatal glutamatergic signaling may represent a pathogenic co-factor for depression in HD [198]. In line with this assumption, a therapeutic intervention aimed at enhancing cystine-dependent synaptic glutamate transporters was able to ameliorate depressive-like behavior in an experimental model of HD [199]. 

### 4.3. Synaptic Dysfunctions, Neuroinflammation, and Psychiatric Disorders

Striatal synaptic dysfunction may also underlie mood symptoms during pathological neuroinflammation. Indeed, an immune-mediated alteration of dopamine neurotransmission in the striatum, with reduced dopamine release and unbalanced signaling through D1- and D2-like receptors, has been linked to depressive-like behavior during experimental MS [200]. Of note, such striatal synaptic and behavioral abnormalities were associated with an increased production of the pro-inflammatory cytokine interleukin 1 beta (IL-1β) in the striatum, and the administration of anti-IL-1β therapy was able to improve mood disturbances in mice with experimental MS [200]. Similarly, anxiety-like behavior during experimental MS has been linked to both an IL-1β-dependent alteration of endocannabinoid and GABAergic striatal signaling [201] and a tumor necrosis factor α (TNF-α)-induced alteration of striatal glutamatergic transmission [202]. It is possible to hypothesize that anxious and depressive symptoms during MS may be triggered by the pathological interaction of proinflammatory molecules with specific synaptic targets and clinical studies support this hypothesis, since CSF levels of pro-inflammatory cytokines, such as interleukin-2 (IL-2), IL-1β and TNF-α, correlated with the degree of anxiety and depressive symptoms in MS patients [203]. 

Interestingly, uncontrolled neuroinflammation may represent a common pathological substrate for further neurological diseases other than MS and psychiatric disorders. The activation of the immune system is known to deeply influence human mood, behavior and cognition, leading to the so-called “sickness behavior”, aimed at re-organizing perceptions and actions of the ill individual in order to better cope with an infectious process and to avoid pathogen spreading in a community [204,205]. This behavioral change is thought to rely on an immune-mediated modulation of synaptic function and could trigger a pathological depressive-like response in disorders characterized by a chronic inflammatory process in the CNS, including neurodegenerative diseases like AD and PD [206,207]. In this context, increased production of TNF-α and decreased levels of the anti-inflammatory mediator transforming growth factor-β (TGF-β) have been considered as neurobiological links between depression and AD [208,209]. 

### 4.4. Future Perspectives

Future translational studies are needed to better dissect the role of synaptopathy in the pathogenesis of psychiatric symptoms during CNS disorders, especially for neurological diseases for which well-defined experimental models are not available, such as FTD. At the same time, from a clinical point of view, a multidisciplinary experimental effort seems to be required to define reliable biomarkers able to reflect the underlying pathogenetic processes.

## 5. Body Fluids Biomarkers and Psychiatric Symptoms in Neurodegenerative and Neuroinflammatory Diseases

Body fluids biomarkers play an emerging role in the early identification of neurological—and especially neurodegenerative—diseases since they can reflect the pathophysiology of CNS disorders. For this reason, they represent a unique tool for better understanding the biological bases of clinical manifestations of neurological diseases, including psychiatric presentations. The proximity of CSF to CNS makes this biofluid the ideal source of biomarkers of the ongoing biological processes. Though the measurement of CNS-derived biomarkers is possible also in blood, blood biomarkers cannot still completely replace CSF biomarkers, which rely on more standardized analytical procedures and on validation in larger cohorts [18]. 

At a neuronal level, psychiatric disorders and neurological diseases share molecular aspects. However, in front of a common molecular basis, psychopathological manifestations benefit from distinct treatment approaches and show different clinical outcomes, according to their occurrence either as psychiatric disorders or as presentation of neurological diseases. Therefore, the exclusion (or the identification) of neurodegenerative or neuroinflammatory diseases as main cause of psychiatric manifestations, or as comorbidity contributing to the clinical picture, is of utmost importance in routine clinical practice.

### 5.1. Biomarkers of Amyloidosis and Tauopathy

AD provides the best example of the application of CSF biomarkers for a biological definition of the disease. The coexistence of CSF decreased β-amyloid_1-42_/β-amyloid_1-40_ (Aβ42/Aβ40) ratio and elevated phosphorylated tau at threonine-181 (p-tau), which reflect amyloidosis and tauopathy respectively, allows to claim the presence of AD-related neurobiology, regardless to cognitive stage and clinical presentation [210]. This supports the potential use of CSF biomarkers to detect (or rule out) an AD signature in subjects with psychiatric presentation. 

In a recent multicenter study carried out in memory clinics, about 20% of patients firstly diagnosed with an idiopathic psychiatric disorder showed, in fact, a CSF AD profile [211]. Findings from population-based cohorts indicated that amyloid-related depression (i.e., depressive symptoms and biomarkers positivity for amyloidosis) resulted in a greater impairment in memory, visuospatial abilities, and executive functions, with respect to non-amyloid-related depression, during a long-lasting follow-up in elderly subjects, also predicting an earlier development of dementia [212,213]. This suggests that depression, as preclinical manifestation of AD, might be strongly associated with amyloid-related pathway. 

Along the pathophysiological cascade of AD, amyloidosis happens first, and it is followed by neurofibrillary tangles deposition [210]. Therefore, the association between affective symptoms and amyloid-related biomarkers suggests that such symptoms represent early manifestations of AD pathology, before significant and extensive tau deposition is found. Accordingly, Aβ biomarkers might be helpful to establish whether elderly individuals showing affective symptoms could be, in fact, in the AD continuum. 

More recently, different investigations focused on blood p-tau as a more easily accessible biomarker reflecting AD pathophysiology. Preliminary findings indicated that blood p-tau shows a stepwise elevation along the AD continuum, being increased even in the preclinical stages, with respect to healthy controls and other neurodegenerative disorders [214]. Thus, high-performance assays targeting blood p-tau could represent the first simple, practical, and non-invasive test to rule out AD in case of pure psychiatric debut. 

### 5.2. Biomarkers of Ubiquitinopathy

Differently from AD, CSF biomarkers reflecting the pathophysiology of other neurodegenerative diseases still lack clinical validation and are mainly used in research settings. TAR DNA binding protein 43 (TDP43), which is primarily involved in alternative splicing and transcriptional regulation, is associated with the pathophysiology of FTD [215]. Increased levels of both CSF and blood TDP43 have been found in FTD [216,217], also correlating with brain TDP43-related pathology burden [218]. Findings deriving from a recent study focusing on subjects with psychiatric disorders during late-life showed higher serum levels of TDP43 in patients with MDD compared to healthy controls, suggesting that some individuals diagnosed with depression could be affected by bvFTD [219]. Depressive symptoms are common onset manifestations of bvFTD, and peripheral TDP43 levels may represent a promising tool to identify depressed patients who, in fact, are in a prodromal stage of FTD. 

### 5.3. Biomarkers of Synucleinopathy

α-synuclein (α-syn), encoded by *SNCA* gene, is a pivotal protein in the neurobiology of PD. Serum and plasma levels of total α-syn (t-α-syn) have been reported to be either higher, lower, or not significantly different in PD patients compared to controls [220,221,222]. Additionally, serum and plasma α-syn concentrations can be influenced by red blood cells (RBCs) that are the major source of α-syn in blood, with even low RBCs contamination resulting in a substantial increase of α-syn in serum or plasma [223]. These issues limit the utility of blood t-α-syn measurement for diagnostic purposes in PD. Otherwise, a few investigations consistently identified higher blood levels of other α-syn species, namely oligomeric α-syn (o-α-syn) and phosphorylated α-syn (p-α-syn), in PD with respect to healthy controls [224]. 

In psychiatric settings, increased levels of *SNCA* mRNA expression in peripheral blood cells were found in patients with MDD, also correlating with the severity of depressive symptoms [225]. Serum levels of α-syn were showed to be higher in MDD patients than in control subjects, as well [226]. The role of α-syn in psychiatric disorders is not clear, yet. However, given the main physiological role of α-syn in vesicles trafficking at a pre-synaptic level, it can be hypothesized that its altered levels in body fluids might reflect the impairment in synaptic functioning and neurotransmitter machinery occurring in psychiatric disorders. Accordingly, altered concentrations of other synaptic proteins such as SNAP-25 were demonstrated in the brain of patients with schizophrenia [227]. In addition to this explanation, α-syn as a biomarker of synucleinopathy could be implicated in the association between psychiatric symptoms and PD, thus representing a potential biomarker to identify those individuals in which depression is a prodromal manifestation of PD. Investigations focusing on CSF α-syn are needed to better elucidate the role of α-syn as biomarker of prodromal PD in patients with psychopathological manifestations. In this scenario, seeding aggregation assays are promising tools. Among them, the Real Time Quaking Induced Conversion (RT-QuIC) has been demonstrated to be capable of detecting pro-aggregating species of α-syn with high specificity and sensitivity in CSF samples from PD patients, even at prodromal stages, i.e., in patients with REM behavioral sleep disorders (RBD) who developed a clinically manifested PD during the follow-up [228]. 

### 5.4. Biomarkers of Neuroinflammation

In the last few years, growing attention has been paid to the contribution of neuroinflammation to psychopathological manifestations in CNS disorders. Among them, MS represents the disease associated with the most identifiable and measurable contribution of immune system activation. As already discussed, depression in MS has been associated with increased levels of TNF-α, interleukin 1β (IL-1β) and interleukin 6 (IL-6) in both CSF and blood [203,229]. Peripheral levels of IL-1β [203], as well as TNF-α and interferon-γ (IFN-γ), were found to be related to the severity of psychiatric symptoms occurring in the context of a clinical relapse [230]. There is also evidence of increased CSF levels of cytokines (IL-6 and IL-8) in psychiatric disorders including schizophrenia and affective disorders [231,232]. Consistently, patients with depression showed increased levels of serum pro-inflammatory cytokines, which were found to decrease to normal concentrations after clinical recovery [233]. 

### 5.5. Neurotransmitter Biomarkers

Biomarkers reflecting alterations in neurotransmitter pathways have been investigated in CNS diseases showing psychopathological manifestations. Low peripheral levels of serotonin and its metabolite 5-hydroxyindolacetic acid were found to correlate with depressive symptoms severity in PD patients [234]. Depressed MS patients showed lower tryptophan levels and increased levels of its metabolite kynurenine in the CSF, with respect to patients without depression [235]. The monoamine hypothesis is one of the most common theories explaining the development of psychopathological manifestations, based on the evidence that the concentrations of monoamines (serotonin, dopamine, and adrenaline) in synaptic gaps are decreased in patients with psychiatric symptoms. Accordingly, changes in CSF monoamines concentrations were also detected in pure psychiatric disorders [236], suggesting that these molecules are altered in subjects with psychiatric manifestations regardless to their origin, and are not accurate biomarkers to differentiate psychiatric disorders and neurological diseases.

### 5.6. Biomarkers of Axonal Damage in the Early Detection of CNS Disorders in Patients with Psychiatric Symptoms

Independently of the underlying etiology, most CNS degenerative and inflammatory diseases are characterized by increased levels of CSF and serum neurofilament light chain (NfL), which is a reliable marker of damage affecting large myelinated axons [237]. Both CSF and blood concentrations of NfL have shown good accuracy to discriminate AD patients from healthy individuals [238,239]. Additionally, NfL levels start to increase in blood more than 15 years before the first clinical manifestation of AD, as demonstrated by longitudinal studies on AD mutation carriers [237], possibly representing a useful marker to detect AD patients with prodromal psychopathological manifestations. With respect to AD, elevation in CSF NfL levels is even more pronounced in FTD [240], in which this biomarker can exert a very relevant clinical application in the differential diagnosis with non-neurodegenerative disease mimics. Preliminary findings indicated an excellent ability of CSF NfL in distinguishing bvFTD from primary psychiatric conditions [237]. In PD patients, it seems that CSF NfL values do not increase, with respect to healthy subjects [237]. Otherwise, elevated CSF and blood NfL concentrations have been described in HD, especially in patients with disease manifestations including psychiatric symptoms, compared with asymptomatic carriers of cytosine-adenine-guanine (CAG) triplet repeats [237]. CSF NfL levels are also increased early in MS since its first clinical presentation, that is, clinically isolated syndrome (CIS) [241]. 

Since NfL is a sensitive but unspecific marker of axonal injury, its diagnostic value does not rely on discriminating between neurological diseases with similar degree of axonal loss, but rather between CNS disorders with different disease intensity or progression rate, and moreover between neurodegenerative/neuroinflammatory and non-neurological diseases. For this reason, in case of psychiatric manifestations, high values of NfL in blood or CSF have been proposed as a red flag alerting the physician about the existence of underlying secondary causes [17]. 

### 5.7. Future Perspectives on Synaptic, Glial and Neurotransmitter Biomarkers

A series of other CSF biomarkers can be considered in the context of neurodegenerative and neuroinflammatory disorders. These include synaptic proteins such as neurogranin and glial activation-related biomarkers, i.e., Chitinase 3-like 1 (YKL40) and soluble form of the triggering receptor expressed on myeloid cells 2 (sTREM2), which have been extensively investigated both in AD and PD [242,243,244]. Different studies support the utility of these newer biomarkers in the diagnostic and prognostic assessment of CNS disorders. However, given the role of synaptopathy and neuroinflammation even in primitive psychiatric diseases, whether these molecules could be also useful to discriminate neurological diseases from pure psychiatric conditions remains an unsolved question. 

## 6. Conclusions

Psychiatric symptoms are highly prevalent in neurodegenerative and neuroinflammatory diseases, being documented at any disease stage. Psychopathological manifestations during CNS disorders can be explained by alterations in specific brain networks. Indeed, structural and functional abnormalities involving brain regions and networks entangled in the emotional-affective regulation, i.e., frontal lobes, basal ganglia, and limbic system structures, have been associated with psychiatric manifestations in CNS diseases. At neuronal level, synaptic transmission and plasticity dysfunctions are involved in the onset of psychopathology in neurodegenerative conditions. Furthermore, the possible role of immune-mediated synaptopathy in the development of psychiatric symptoms in CNS disorders has recently been documented. In some cases, psychiatric symptoms may represent the early expression of pathophysiological mechanisms that also contribute to the onset of neurological manifestations. 

Given these premises, the exclusion of a neurological condition should be mandatory in the diagnostic work-up of psychopathological manifestations, especially when they begin at advanced ages. The systematic investigation of an organic cause of psychiatric symptoms represents a methodological approach that further improves the possibility for an early diagnosis and appropriate treatment of CNS disorders. In this scenario, body fluids biomarkers able to detect an underlying neurological disease play an emerging role to support the diagnosis of such disorders even in case of a pure psychiatric presentation. Some of these biomarkers, like those related to amyloidosis and tauopathy, are already used in routine clinical practice for diagnosing AD even in case of atypical presentations of the disease. Other biomarkers such as those associated with α-syn and TDP43 pathologies, though promising, need to be validated for diagnostic purposes in PD and FTD, respectively, before they can be reliably investigated to detect these diseases in case of psychiatric manifestations. In turn, research on body fluids biomarkers in psychiatric disorders could be of added value to refine the pathophysiological underpinnings of these conditions. Further knowledge in this field may help to better characterize psychiatric symptoms in neurological diseases, possibly predicting their onset during the disease course and assisting in finding effective therapies. This may be particularly relevant since psychiatric symptoms in neurological diseases have a significant impact on patients’ quality of life and clinical outcome. To this aim, a series of pre-analytical and analytical issues need to be addressed. Thus, longitudinal studies should be carried out to test the capacity of these biomarkers in characterizing the pathophysiological correlates of psychiatric symptoms from early to advanced stages of neurological diseases. Further biomarkers, such as those related to multimodal imaging, neuronal oscillations, or genetics should also be considered in future research, in order to provide a more comprehensive knowledge of the complex pathophysiological bases of psychiatric symptoms in CNS disorders as well as to define their potential role in the prediction of development and progression of such alterations.

Moreover, the further characterization of pathophysiological mechanisms underpinned by psychiatric symptoms in CNS diseases may increase our knowledge regarding the diagnosis and treatment of psychiatric disorders in the future, even when they do not co-occur with neurological diseases. Indeed, the description of specific brain systems that may be disrupted, as well as the detection of possible molecular correlates, could help overcoming traditional psychiatric diagnostic categories towards a precision psychiatry perspective. This could allow to address better treatment strategies in psychiatry, which may also encompass lifestyle and dietary interventions.

In conclusion, greater awareness should be dedicated to the possible co-occurrence of psychiatric symptoms in CNS disorders in the clinical practice. Indeed, psychiatrists and neurologists should refer to a common background under a clinical and biological point of view, thus implementing a shared diagnostic and therapeutic management of CNS disorders in case of a psychiatric presentation.
